# Solitary metastatic adenocarcinoma of the sternum treated by total sternectomy and chest wall reconstruction using a Gore-Tex patch and myocutaneous flap: a case report

**DOI:** 10.1186/1752-1947-4-75

**Published:** 2010-03-01

**Authors:** Stavros I Daliakopoulos, Michael N Klimatsidas, Reiner Korfer

**Affiliations:** 1 Herz-und Diabeteszentrum Nordrhein Westfalen, Georgstrasse 11, Bad Oeynhausen, Universitätsklinikum der Ruhr-Universität Bochum, Germany; 2 Glenfield Hospital, Cardiothoracic Surgery Department, University Hospital of Leicester, UK

## Abstract

**Introduction:**

The consequences of bone metastasis are often devastating. Although the exact incidence of bone metastasis is unknown, it is estimated that 350,000 people die of bone metastasis annually in the United States. The incidence of local recurrences after mastectomy and breast-conserving therapy varies between 5% and 40% depending on the risk factors and primary therapy utilized. So far, a standard therapy of local recurrence has not been defined, while indications of resection and reconstruction considerations have been infrequently described. This case report reviews the use of sternectomy for breast cancer recurrence, highlights the need for thorough clinical and radiologic evaluation to ensure the absence of other systemic diseases, and suggests the use of serratus anterior muscle flap as a pedicle graft to cover full-thickness defects of the anterior chest wall.

**Case presentation:**

We report the case of a 70-year-old Caucasian woman who was referred to our hospital for the management of a retrosternal mediastinal mass. She had undergone radical mastectomy in 1999. Computed tomography and magnetic resonance imaging revealed a 74.23 × 37.7 × 133.6-mm mass in the anterior mediastinum adjacent to the main pulmonary artery, the right ventricle and the ascending aorta. We performed total sternectomy at all layers encompassing the skin, the subcutaneous tissues, the right pectoralis major muscle, all the costal cartilages, and the anterior part of the pericardium. The defect was immediately closed using a 0.6 mm Gore-Tex cardiovascular patch combined with a serratus anterior muscle flap. Our patient had remained asymptomatic during her follow-up examination after 18 months.

**Conclusion:**

Chest wall resection has become a critical component of the thoracic surgeon's armamentarium. It may be performed to treat either benign conditions (osteoradionecrosis, osteomyelitis) or malignant diseases. There are, however, very few reports on the results of full-thickness complete chest wall resections for locally recurrent breast cancer with sufficient safety margins, and even fewer reports that describe the operative technique of using the serratus anterior muscle as a pedicled flap.

## Introduction

Bone metastasis is a frequent complication of cancer. It occurs in up to 70% of patients with advanced breast or prostate cancer and in approximately 15% to 30% of patients with carcinoma of the lung, colon, stomach, bladder uterus, rectum, thyroid or kidney. Breast cancer has the tendency to relapse in the bones, and 56% of autopsy cases reveal the occurrence of bone metastasis. The most frequent sites of bone metastasis are the thoracic and lumbosacral spine. The consequences of bone metastasis are often devastating, as only 20% of patients with breast cancer are still alive five years after the discovery of bone metastasis. Chest wall resection for breast cancer was first performed by Schede in 1866 and then by Sauerbruch in 1907. Meanwhile, partial sternectomy for a primary sarcoma was first described by Holden in 1878. In 1959, Brodin and Linden first performed and described total sternectomy due to chondrosarcoma involving the entire sternum.

The surgical treatment of chest wall tumors challenges the aggressiveness and ingenuity of the operating surgeon who closes the defect. Partial or total sternectomy, together with rib resection, are common thoracic surgical procedures. These are undertaken for primary and secondary tumors arising from any of the structures forming the chest wall, as well as recurrent breast cancer or lung tumors invading the chest wall. Myocutaneous flaps and prosthetic materials greatly facilitate reconstruction after massive chest wall resection.

## Case presentation

We report the case of a 70-year old Caucasian woman who was referred to the thoracic oncology unit of our hospital for the management of a retrosternal mediastinal mass. She had been well 8 weeks before admission when she experienced the sudden onset of sharp left anterior chest pain. The pain was worse in the area adjacent to the sternum and also worsens when she takes a deep breath. In 1999, she underwent radical left side mastectomy followed by CEF (cyclophosphamide, epirubin and fluorouracil) chemotherapy and radiotherapy. The clinical and histological characteristics of the primary breast cancer revealed a Stage IIIa adenocarcinoma with positive axillary lymph node metastasis. Her estrogen receptor assay, as well as the amplification of the human epidermal growth factor receptor type 2 (HER 2/neu) was negative. Expression of her progesterone receptors was defined as low (Reiner score for staining of tumour-cell nuclei). On admission our patient's vital signs were normal. She had neither jugular venous distension nor cervical or supraclavicular lymphadenopathy. Her chest was clear on auscultation. There was no tenderness on palpation of her ribs or sternum. The remainder of her examination results was normal.

**Figure 1:**
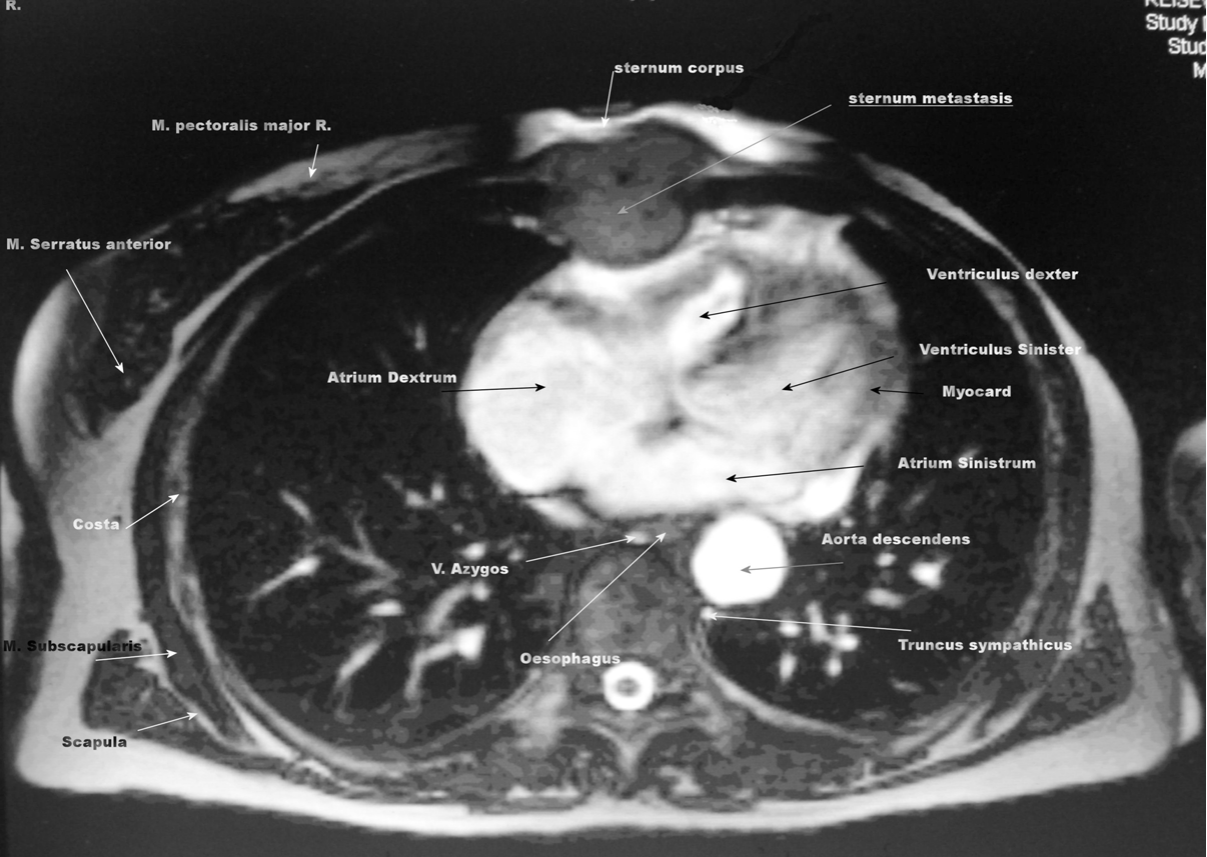
**Magnetic resonance imaging of axial plan with intravenous contrast gadolinium-BOPTA demonstrating a mass adherent to the right ventricular wall**.

**Figure 2:**
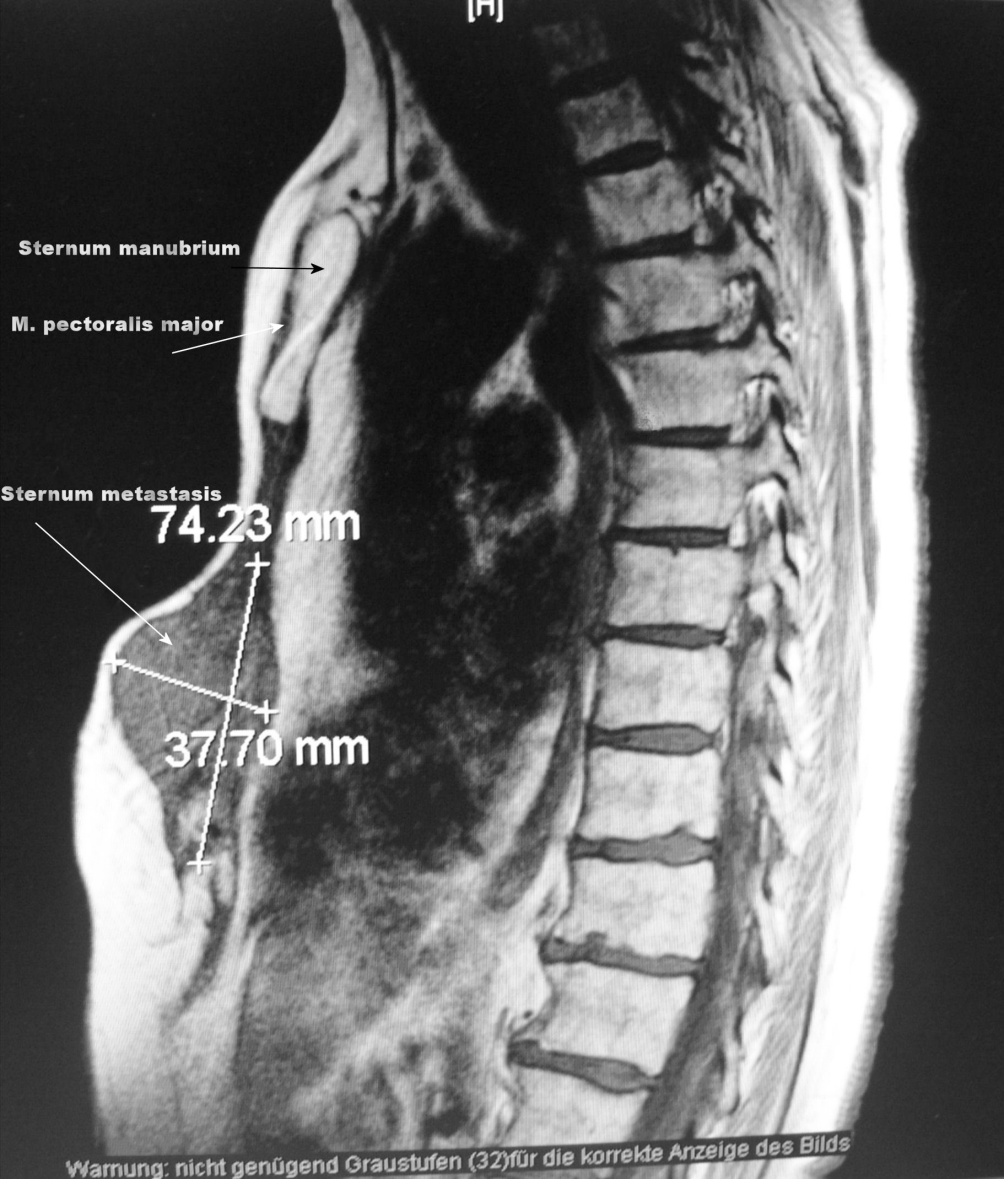
**Magnetic resonance imaging of the sagittal plan of the large adenocarcinoma**. Whole thickness invasion of the sternum, surrounding fat tissues, and the anterior mediastinum is shown.

Computed tomography (CT) scanning and magnetic resonance imaging (MRI) of our patient's chest revealed a 74.2 × 37.7 × 133.6-mm mass in her anterior mediastinum adjacent to the main pulmonary artery, the right ventricle, and ascendens aorta contiguous to the pericardium (Figures 1 and 2). There was no specific direct evidence of vascular invasion but we raised the question of pericardial invasion. Our patient was vigorously scrutinized for metastatic disease, which included routine blood chemistries and the determination of lipid associated sialic acid, carcinoembryonic antigen (CEA) and CA 15-3 serum markers. CT scan of her neck and abdomen for restaging revealed no other foci of metastatic disease. A bone scan showed uptake only in her sternum.

Our patient was anaesthetized and ventilated with a double-lumen endotracheal tube. An epidural catheter was also inserted for pain control during the peri-operative period. Our patient was placed at first in a right thoracotomy position with soft rotation of the coxa towards the surgeon. Standard thoracotomy incision was used to expose the serratus anterior muscle (SAM). The SAM was identified and mobilized by separating it from the chest wall and then carefully dividing its attachments to the first 4 to 5 ribs, with a periosteal elevator at first and then with a cautery. As dissection proceeded upward toward her axilla, the contribution of the lateral thoracic artery was seen entering the muscle on its anterior cephalic border. The blood supply to the serratus anterior may come from the thoracodorsal pedicle, from the subscapular pedicle, or directly from the axillary artery. In our case, our patient's blood supply came from the serratus anterior branch from the thoracodorsal artery, which originates as the largest branch from the subscapular artery. During the procedure these branches were identified and preserved. The harvested SAM was advanced and transposed within its arc of rotation towards the midsternal line to cover the defect.

**Figure 3:**
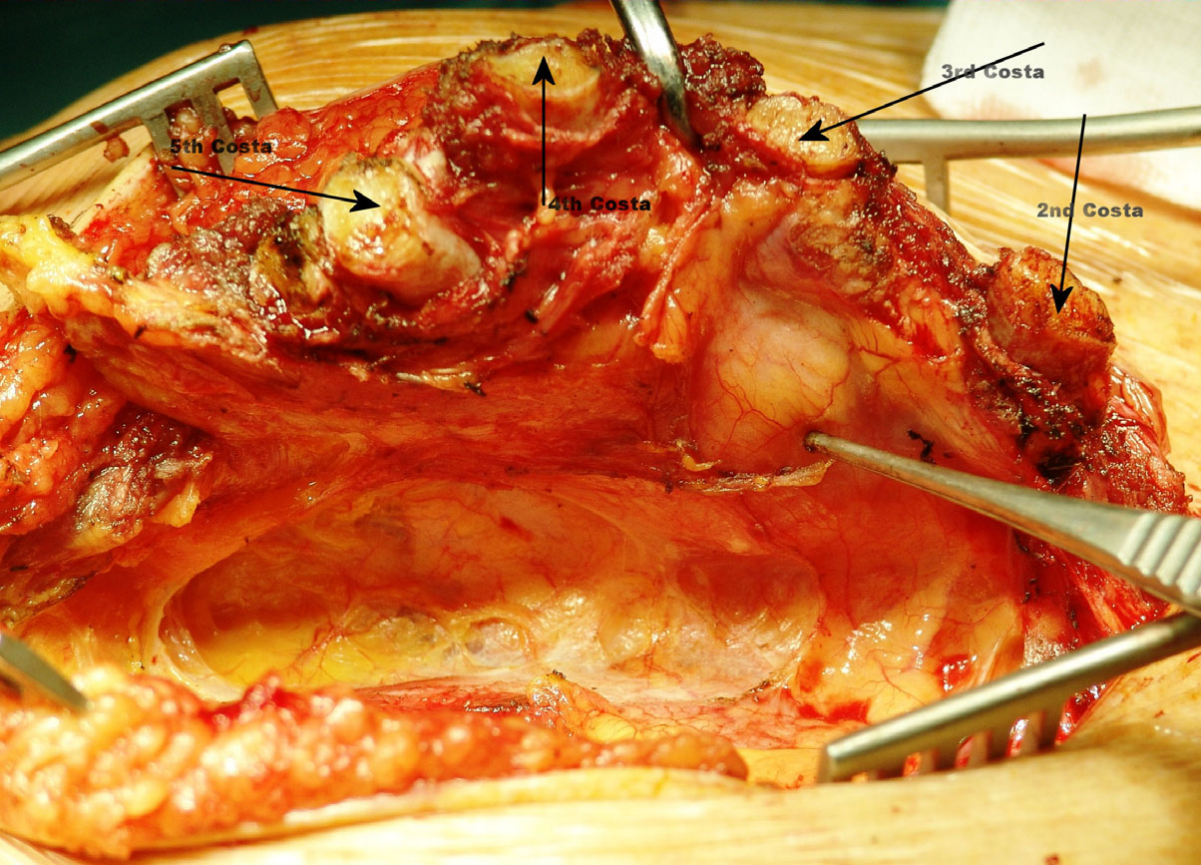
**Operative view of the adenocarcinoma**. Arrows indicating costal cartilages of the first ribs.

**Figure 4:**
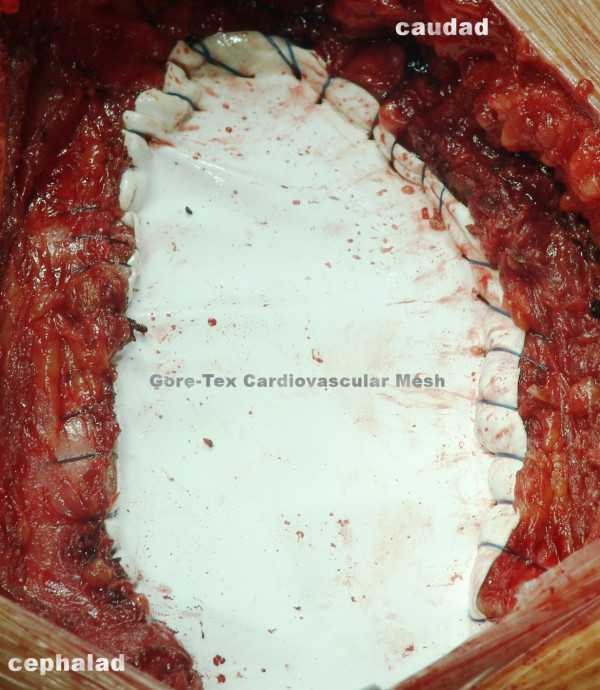
**Immediate closure of the defect with the Gore-Tex mesh**.

**Figure 5:**
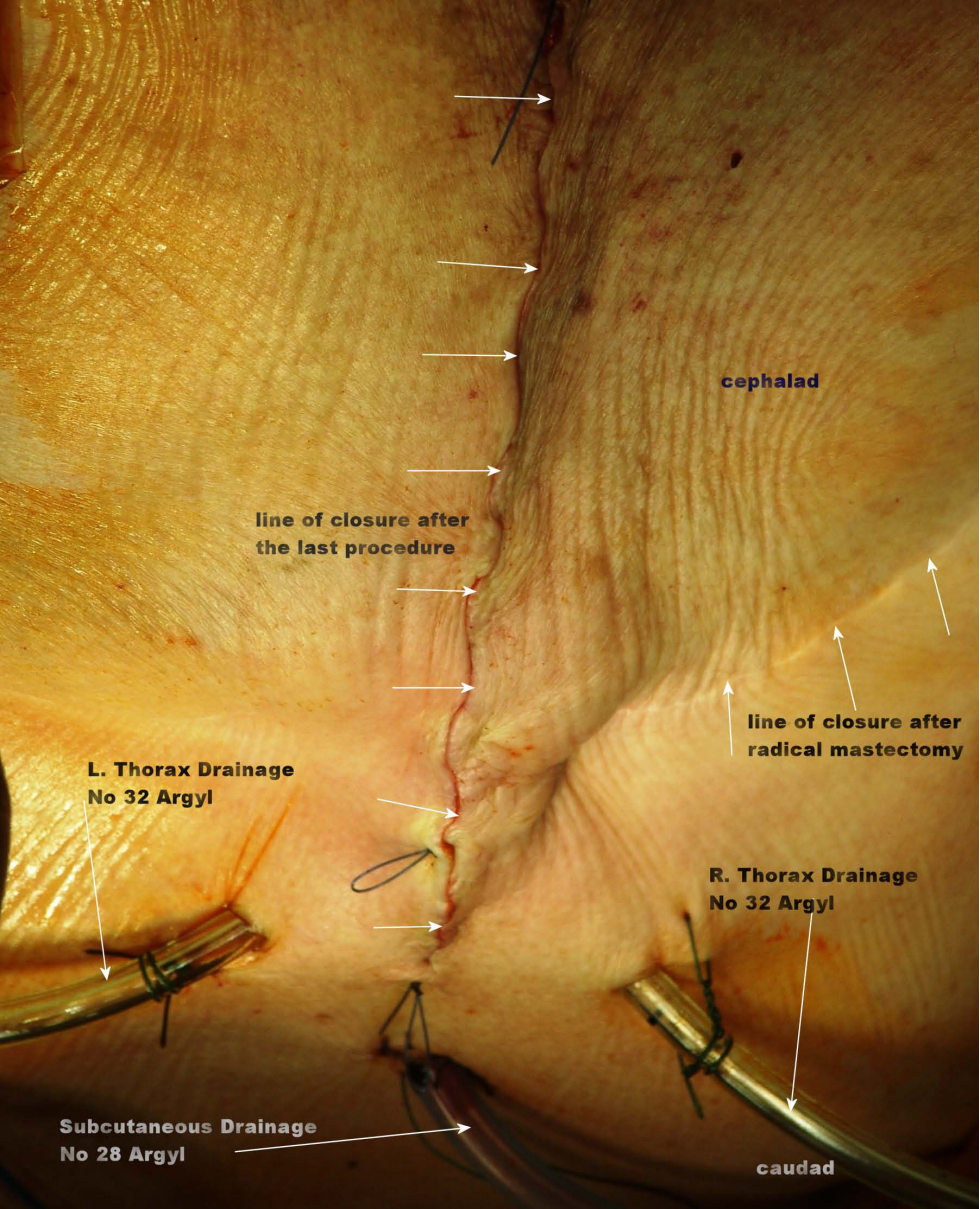
**Immediate closure of the skin after the end of the procedure**.

The sternal incision started at the level of our patient's manubriosternal joint and extended inferiorly to her xiphisternum. The surgical resection was a vertical elliptical incision of the visible mass. Total sternectomy was performed at all layers encompassing our patient's skin, subcutaneous tissues, right pectoralis major muscle, all her costal cartilages of the first five ribs (Figure 3), and the anterior part of her pericardium. Mobilization began first on one side of her sternum with exposure and section of the ribs. The ribs were divided laterally. Her right internal thoracic artery and the intercostals neurovascular bundle were ligated with absorbable suture. Lastly, the critical point of mass attachment to the heart was approached. Immediate closure of the defect was performed without cement but with a single 0.6-mm Gore-Tex cardiovascular mesh (W. L. Gore and Associates, Flagstaff, Arizona) which was cut to a size smaller than that of the defect. The mesh was thus effectively stretched when it was sutured to her chest wall so that any laxity in the reconstruction was alleviated (Figures 4 and 5). The serratus anterior myocutaneous flap was then secured with heavy non-absorbable suture over the Gore-Tex mesh.

**Figure 6:**
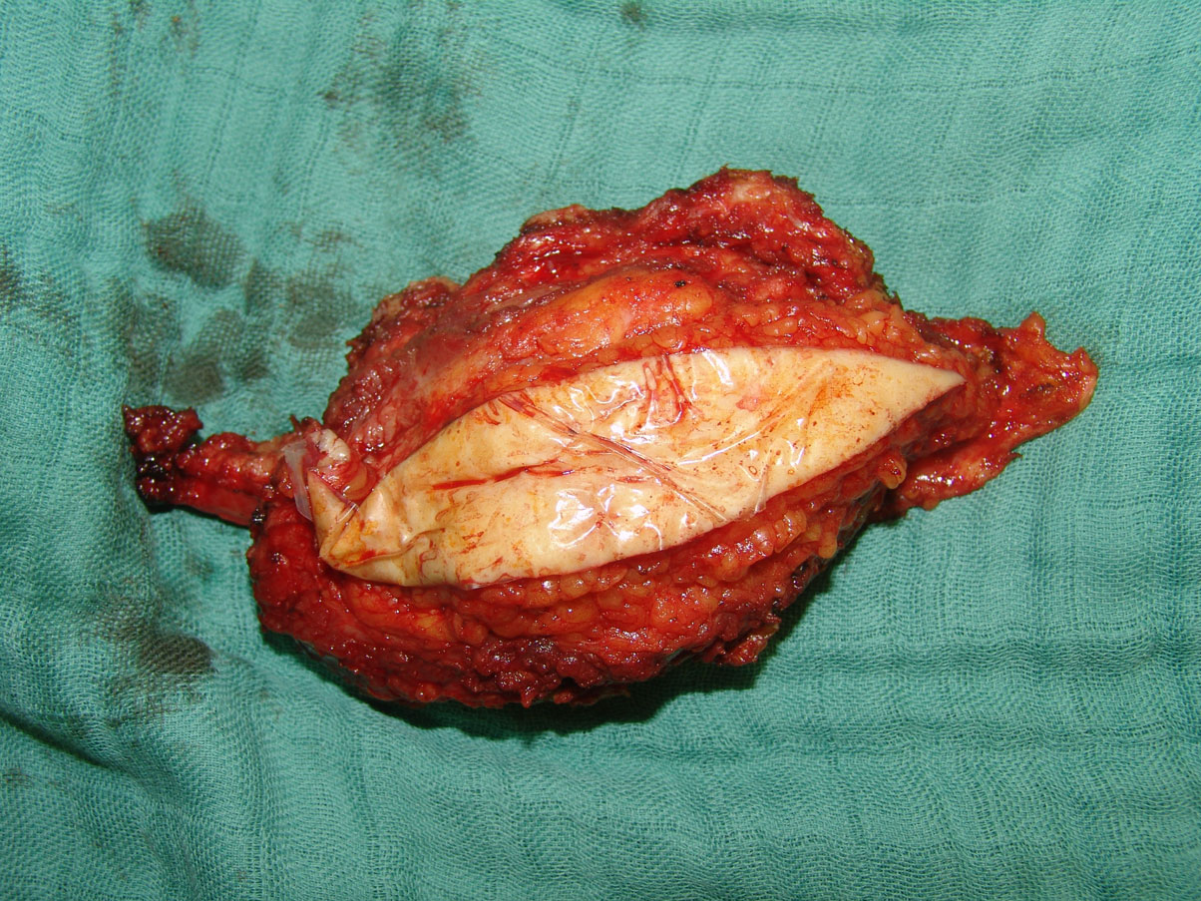
**The mass**.

Pathology revealed a poorly differentiated invasive carcinoma infiltrating our patient's sternum with the involvement of the pericardium (Figure 6). The amplification of the human factor margins was clear, while immunohistochemistry was negative.

Epidural analgesia was employed in the immediate postoperative period. Postoperative respiratory function tests revealed satisfactory results and our patient could be relieved from endotracheal intubation a day after the operation. She did not have any problems in her daily activities or any occurrence of chest flailing or paradoxical movement of the chest. Scapular winging occurred although any effort was made to preserve the a third of the lower part of her serratus anterior muscle. No flap infection or wound dehiscence was noted, and she was discharged from the hospital nine days after the operation.

She received two additional cycles of CEF chemotherapy consisting of 2 cycles of oral cyclophosphamide at a dose of 75 mg/m^2 ^on days 1 through 14, 60 mg/m^2 ^of epirubicin on days 1 and 8, and 500 mg/m^2 ^of fluorouracil intravenously on days 1 and 8. During her CEF therapy she also received antibiotic prophylaxis with ciprofloxacin at a dose of 500 mg orally twice daily. She is well 18 months after the diagnosis, and she exhibited no evidence of recurrent disease on serial CT and MRI scans of her chest, abdomen, and brain. She remains asymptomatic and the stability of her chest wall is well-preserved.

## Discussion

The operative management of massive chest wall malignancies presents as an infrequent but formidable surgical challenge mainly because of the difficulty in making full thickness resections without compromising the stability and the reconstruction of the chest wall. A review of the literature showed that a complete chest wall resection is only performed in very rare cases, with the largest reported study in the last 20 years coming from Mora *et al. *and including 69 patients [1]. In patients with breast cancer, the presence of either sternal involvement or an isolated sternal metastasis is relatively uncommon, with reported incidences of 5.2% and 1.9% to 2.4%, respectively [2]. Sternal involvement may occur either from direct invasion by enlarged internal mammary lymph nodes or from hematogenous spread. However, in contrast with vertebra lesions, which tend to result in multicentric bony disease from spread through the paravertebral plexus [3], some sternal lesions have been observed to remain solitary over time and may be amenable to surgical resection with curative intent [4]. Although local recurrence after breast surgery does not consistently represent systemic metastasis [5], the role of surgery is controversial in breast cancer metastasis involving the thoracic wall and the sternum [6], as well as in sternectomy for isolated breast cancer. This can be gleaned from the fact that the literature consists predominantly of retrospective case series.

Meanwhile, local recurrence following the primary treatment of breast cancer ranges from less than 5% for stage I to greater than 25% for stages II and III with an extremely variable disease-free interval [7]. Since chest wall recurrence is associated with disseminated metastasis in 60% to 100% of cases, simple excision, radiation therapy and chemotherapy are utilized to treat local and systemic diseases. Noguti *et al. *[8] performed sternal resections with parasternal and mediastinal lymph node dissection on nine patients before chemo-endocrine therapy was undertaken. The eventual relapse of the cancer in 8 patients revealed that lymph node dissection had no effect on locoregional control. Nevertheless, dissection provided prognostic information because all patients with involved parasternal and mediastinal lymph nodes relapsed and died within 30 months, while 3 patients without lymph node involvement survived for more than 6 years.

Lequaglie *et al. *performed radical, curative-intent sternectomies in a subgroup of 28 patients with isolated breast cancer recurrence and noted that the 10-year overall survival in the group was 41.8% [9]. Meanwhile, McCormack *et al. *noted in a series of 35 patients that 20 (57.14%) were alive from 5 to 120 months with a median of 50 months [10]. These authors stated that surgical resection of recurrent mammary carcinoma resistant to all other therapy is a viable alternative for palliation and cure in patients who were carefully selected.

Furthermore, Avital *et al. *[11] presented two patients with isolated sternal metastasis from breast cancer that underwent sternectomy followed by systemic chemotherapy and irradiation. Follow-up examinations continued for 30 and 36 months and they were alive and living a good quality of life during this period. One of them, however, had local recurrence in the axilla, but this was resected successfully. Meanwhile, in a series of 100 patients, Brower *et al. *[12] observed that the incidence of local recurrence after radical chest wall resection was 20%, while the incidence of systemic recurrence after chest wall resection was 60%. The mean survival for the entire group was 17 months after chest wall recurrence and radical resection.

On the other hand, Kwai *et al. *stated that an isolated sternal metastasis should be regarded with caution because it is more likely to herald systemic disease than to develop as solitary sternal disease [2]. The authors demonstrated that 54% of patients with breast cancer and solitary sternal disease developed other foci of distant disease within 20 months. The predominance of pulmonary metastasis and distant skeletal disease found in their study was attributed to the drainage of the internal mammary nodes into the subclavian vein. Moreover, Park and Tarver reported 3 cases of solitary sternal metastasis from breast carcinoma that was treated with systemic therapy [13]. Although follow-up results on these patients were not clearly mentioned, the authors stated that single metastasis in the sternum have the unique tendency to remain solitary for longer than metastasis to other sites. According to McKenna *et al.*, even given the advances in the treatment of locally recurrent and advanced breast cancer, 50% to 70% of patients will still succumb to their disease [14].

Mortality after chest wall resection is reported to be 1.6% to 4.5% [15]. The choice of surgical technique depends on a number of factors, of which the most important is the size and site of the lesion.

There is a considerable discussion as to whether the missing bony thorax should be reconstructed [16]. The decision not to reconstruct the skeleton depends on the size and location of the defect, the presence of wound infection, and whether or not the tumour had been previously irradiated. Generally, lesions less than 5 cm in size in any location and up to 10 cm in posterior size need not require functional reconstruction.

Various techniques have been used to repair the defects in the anterior thoracic wall, such as fascia lata, rib grafts, large skin flaps, the contralateral breast, myocutaneous flaps, and various types of prosthetic materials (polypropylene and Vicryl nets, Gore-Tex patches). The use of prosthesis has not been reported to increase septic complications or foreign body reactions [17]. The numerous advances in chest wall reconstruction over the years, including the use of muscle transposition and musculocutaneous flaps, have made these techniques the mainstay in chest wall reconstruction [18]. Gore-Tex has the advantage of being impermeable to air and liquids and provides excellent results in terms of stability, intrathoracic organ protection, and pulmonary expansion [9].

## Conclusions

The most important task as a thoracic surgeon assessing a patient with a solitary metastatic carcinoma of the sternum is to determine the tumor's likelihood of recurrence after surgery and its amenability to a complete resection. The extensive literature on relapsed breast cancer demonstrates that patients with bone metastasis coincident with the initial presentation of their breast cancer have the best outlook, while histological grade and type are the next most important prognostic factors. Patients with grades I and II ductal or lobular cancers have better prognosis than those with grade III tumors. Estrogen receptor positivity, a long disease-free interval (>3 years versus <3 years) and a pre-menopausal status are other factors that predict a longer survival of patients. In the case of our patient, the recurrence after 9 years following mastectomy, chemotherapy and radiotherapy led us to treat the sternal metastasis aggressively.

The localization of the probable defect after resection, its depth, width, convenient tissue flaps, and the tissue amount necessary for reconstruction must be evaluated pre-operatively. The serratus anterior muscle is a reliable muscle flap with a consistently long pedicle and excellent malleability, thus permitting the coverage of complex three dimensional wounds. It has been successfully used for flap reconstruction of the lower limps, dorsal surface hand defects, injuries to the head, neck and extremities, as well as bony and soft tissue defects in the face. There are only a few cases of flap reconstruction in relation to anterior thoracic wall defects [18,19].

Metastatic breast cancer confined to the skeletal system is a complication that can be diagnosed relatively easily. It is highly responsive to treatment and it is frequently associated with extended patient survival [20]. As our experience in solid recurrent breast cancer sternal metastasis teaches, full thickness chest wall resection remains integral in controlling major complications associated with the chest wall reconstruction because it improves the quality of our patient's life, may provide patients with durable disease-free remission, and can improve survival with low mortality and morbidity results.

## Consent

Written informed consent was obtained from our patient for publication of this case report and any accompanying images. A copy of the written consent is available for review by the Editor-in-Chief of this journal.

## Competing interests

The authors declare that they have no competing interests.

## Authors' contributions

SID participated in sequence alignment, designing the case report and drafting the manuscript. MNK participated in the design of the case report and culled relevant information. RK coordinated the preparation of the case report and designed the whole manuscript. All authors read and approved the final manuscript.
